# Glaucocalyxin A Ameliorates Hypoxia/Reoxygenation-Induced Injury in Human Renal Proximal Tubular Epithelial Cell Line HK-2 Cells

**DOI:** 10.3390/ijms23010446

**Published:** 2021-12-31

**Authors:** Keiko Hosohata, Denan Jin, Shinji Takai

**Affiliations:** 1 Education and Research Center for Clinical Pharmacy, Osaka Medical and Pharmaceutical University, Osaka 569-1094, Japan; 2 Department of Innovative Medicine, Osaka Medical and Pharmaceutical University, Osaka 569-1094, Japan; denan.jin@ompu.ac.jp (D.J.); shinji.takai@ompu.ac.jp (S.T.)

**Keywords:** ischemia-reperfusion injury, reactive oxygen species, protective effects, natural products, glaucocalyxin A

## Abstract

Ischemia-reperfusion injury is one of the major causes of acute kidney injury (AKI), which is increasingly prevalent in clinical settings. Glaucocalxin A (GLA), a biologically ent-kauranoid diterpenoid, has various pharmacological effects like antioxidation, immune regulation, and antiatherosclerosis. In this study, the effect of GLA on AKI and its mechanism were studied in vitro. HK-2 human renal tubular epithelial cells were exposed to hypoxia/reoxygenation (H/R), which were established as an in vitro AKI model. Subsequently, the mRNA expressions of inflammatory and antioxidant factors were determined by quantitative reverse transcription polymerase chain reaction (RT-qPCR). Reactive oxygen species (ROS) production and cell death were detected by fluorescence-activated cell sorting. GLA pre-treatment improved the cell viability of HK-2 cells exposed to H/R. GLA suppressed the H/R-induced ROS production in HK-2 cells. GLA also elevated the activities of superoxide dismutase of HK-2 cells exposed to H/R. Moreover, GLA prevented H/R-induced cell death in HK-2 cells. Furthermore, GLA ameliorated the activation of the protein kinase B (Akt)/nuclear factor erythroid 2-related factor 2 (Nrf2)/heme oxygenase-1 (HO-1) signaling pathway in HK-2 cells exposed to H/R. Our findings suggested that GLA protected HK-2 cells from H/R-induced oxidative damage, which was mediated by the Akt/Nrf2/HO-1 signaling pathway. These results indicate that GLA may serve as a promising therapeutic drug for AKI.

## 1. Introduction

Glaucocalyxin A (GLA) is a natural diterpenoid compound isolated from *Rabdosia japonica* (Burm. f.) var. *glaucocalyx* (Maxim.) Hara, which is widely distributed in East Asia. GLA has been used from a long time ago as a traditional medicine for gastrointestinal disorders and inflammation [1]. Recently, it has been reported that GLA inhibits various pharmacological effects. For example, GLA exhibits anti-cancer [2], antioxidative [3], anti-inflammatory [4], and anti-fibrotic [5] effects. GLA has been shown a possible potential therapeutic agent for microglia-mediated neuroinflammatory diseases via inhibition of the NF-κB and p38 signaling pathways [4]. Furthermore, GLA has been shown to reduce lipopolysaccharide-induced septic shock and inflammation via suppressing the activation of NLRP3 and NLRC4 inflammasomes. There is accumulating evidence about anti-inflammatory effects on many organs such as the nerves [4], liver [3], bone [6], and lung [7]. However, there are a few reports about the ameliorating effects of GLA on kidney injuries.

Acute kidney injury (AKI) is a serious problem in clinical settings [8], which contributes to the development of chronic kidney disease (CKD) and end-stage kidney disease (ESKD) [9,10]. Ischemia-reperfusion (I/R) injury in the kidney is the most common cause of AKI, and a variety of pathological processes and mediators are involved in the I/R-related injury, including generation of reactive oxygen species (ROS), inflammatory response, endoplasmic reticulum stress, and intracellular calcium overload [11,12]. Among these, overproduction of ROS is the major intracellular event during I/R injury. ROS plays an important role in maintaining an intracellular signal system under normal conditions, but the amount of ROS increases markedly in the pathologic state, which causes oxidative stress contributing to renal injury and cell death through a variety of mechanisms, such as inducing the activation of proapoptotic pathways. Therefore, attenuating I/R-mediated oxidative stress and apoptosis may be beneficial for preventing AKI.

Here, we explored the effect and mechanism of GLA on renal I/R injury using renal tubular cell line, HK-2 cells.

## 2. Results

### 2.1. GLA Ameliorates Cell Viability of HK-2 Cells under H/R Stimulation

**Figure 1:**
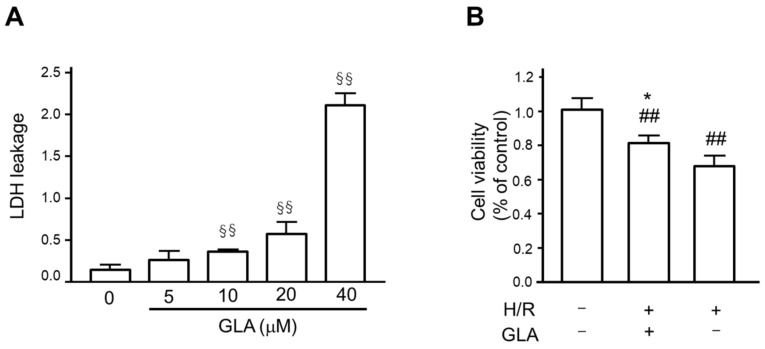
GLA ameliorated cell viability of HK-2 cells. (**A**) To determine GLA concentration, we assessed the cytotoxicity of GLA on HK-2 cells after incubation with a series concentration of GLA (0, 5, 10, 20, and 40 μM) for 24 h using an LDH assay. LDH leakage was calculated as a fraction of the total LDH activity. ^§§^ *p* < 0.01 vs. control. (**B**) To assess the protective effect of GLA on the cell viability of HK-2 cells exposed to H/R, we conducted the MTT assay. The cells were pre-treated with 5 μM GLA for 2 h and then exposed to H/R for 24 h. ^##^ *p* < 0.01 vs. control (normoxia). * *p* < 0.05, vs. H/R. Data are shown as the means ± SE for six wells. GLA: glaucocalyxin A; H/R: hypoxia/reoxygenation; LDH: lactate dehydrogenase.

First, we assessed the cytotoxic effects of GLA on HK-2 cells using an LDH leakage assay. The cells were incubated with a series concentration of GLA (0, 5, 10, 20, and 40 μM) for 24 h. As shown in Figure 1A, GLA at the concentration of more than 10 μM caused a significant increase in LDH leakage, whereas the concentration of GLA at 5 μM showed no significant cytotoxicity itself following 24 h exposure. Then, we determined the GLA concentration of 5 μM in the following experiments. Next, we examined the effect of GLA on cell viability under H/R exposure. The results of the MTT assay showed that H/R exposure significantly decreased viability of HK-2 cells compared with control (normoxia), which ameliorated by pre-incubation with GLA 5 μM (pre-treatment of GLA followed by H/R vs. H/R) (Figure 1B).

### 2.2. GLA Alleviates Oxidative Stress in HK-2 Cells under H/R Exposure

**Figure 2:**
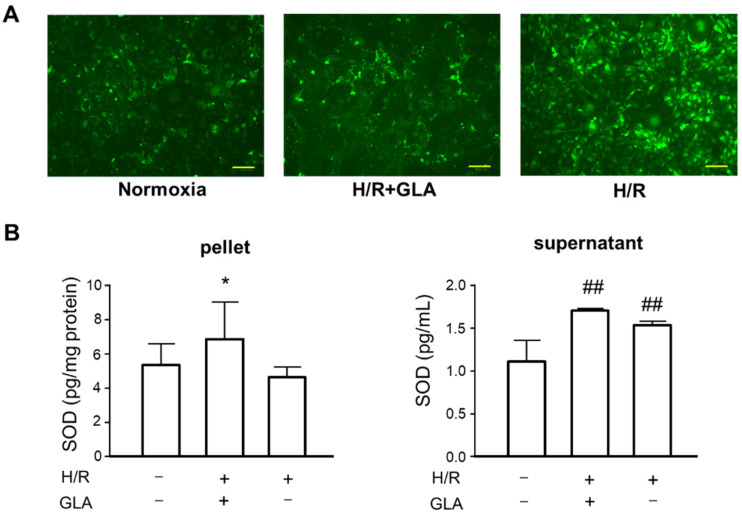
GLA inhibited H/R-induced oxidative stress in HK-2 cells. The cells were pre-treated with 5 µM GLA for 2 h and then exposed to H/R for 24 h. (**A**) STAINING of ROS production in HK-2 cells using DCFH-DA. Scale bars = 100 µm. (**B**) ELISA was conducted to assess the SOD activity in HK-2 cells. ^##^ *p* < 0.01, vs. control (normoxia); * *p* < 0.05, vs. H/R. DCFH-DA: 2’-7’ dichlorofluorescin diacetate; GLA: glaucocalyxin A; H/R: hypoxia/reoxygenation; ROS: reactive oxygen species; SOD: superoxide dismutase. Data are shown as the means ± SE for six wells.

To elucidate the protective effect of GLA against H/R injury in HK-2 cells, we investigated the possible mechanisms in vitro. Figure 2A shows that H/R markedly induced ROS generation in HK-2 cells as compared with control (normoxia), whereas the production of ROS was attenuated by GLA. In addition, GLA significantly increased superoxide dismutase (SOD) in the pellet of HK-2 cells exposed to H/R. Similarly, the GLA increased the activities of SOD. These data demonstrated that GLA alleviated H/R-triggered oxidative stress in HK-2 cells.

### 2.3. GLA Activates Akt/Nrf2/HO-1 Signaling Pathway in HK-2 Cells Exposed to H/R

**Figure 3:**
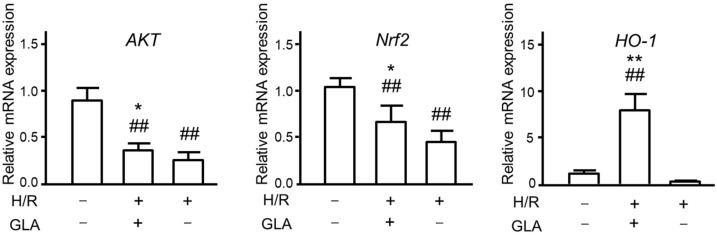
Effects of GLA on oxidative stress in HK-2 cells exposed to H/R. The cells were pre-treated with 5 μM GLA for 2 h and then exposed to H/R for 24 h. ^##^ *p* < 0.01, vs. control (normoxia); * *p* < 0.05; ** *p* < 0.01, vs. H/R.

Next, we explored further mechanisms underlying the protective effect of GLA against HR-induced oxidative stress. As several studies reported that oxidative stress is associated with Akt/Nrf2/HO-1 pathway [13,14,15], we investigated whether GLA could affect the mRNA expression of these oxidative stress-associated proteins. As indicated in Figure 3, H/R exposure significantly downregulated the mRNA expression of Akt and Nrf2 compared with control (normoxia); however, GLA treatment restored the decreased expressions of Akt/Nrf2. Of note, HO-1 mRNA expression was markedly and significantly elevated by GLA treatment in HK-2 cells exposed to H/R.

### 2.4. GLA Attenuates H/R-Induced Cell Death in HK-2 Cells

**Figure 4:**
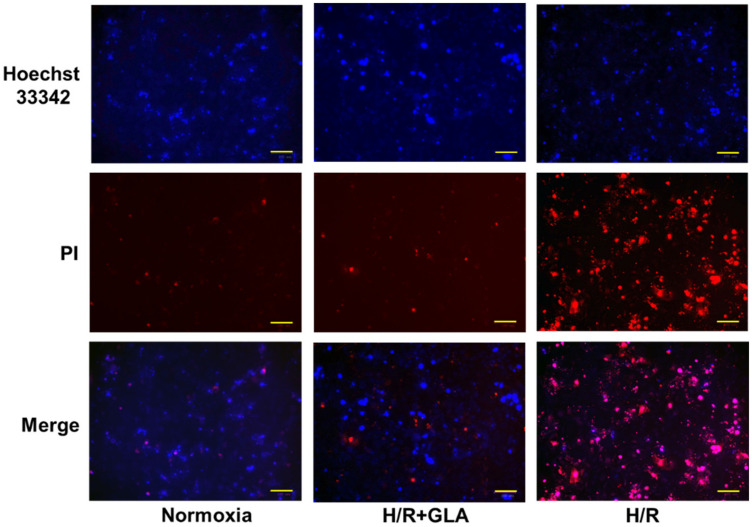
Effects of GLA on H/R-induced cell death in HK-2 cells. The cells were pre-treated with 5 µM GLA for 2 h and then exposed to H/R for 24 h. Cells stained with Hoechst 33342 (blue) and propidium iodide (PI, red) dyes were observed and photographed with a fluorescence microscope. Scale bars = 100 μm.

Subsequently, cell death was assessed in Figure 4. H/R induced an obvious increase in PI-positive cells; however, GLA pre-treatment suppressed PI-positive cells under H/R exposure. Then, GLA pre-treatment inhibited the death of HK-2 cells (Figure 4).

## 3. Discussion

In the present study, we firstly investigated the inhibitory effect of GLA on H/R-induced renal injury, which is one of the most familiar models of AKI in vitro. Our results revealed that GLA significantly inhibited H/R-induced oxidative stress, regulated the mRNA expressions of markers involved in oxidative damage, and increased cell viability under H/R exposure. Furthermore, we found that its anti-inflammatory effects were associated with the activation of the Akt/Nrf2/HO-1 signaling pathway in human renal tubular cell line HK-2 cells. Our study indicated that GLA might be a potential therapeutic agent for AKI treatment.

According to previous studies, GLA has been demonstrated to show suppressive effects on osteoclastogenesis by RANKL [6], inflammatory responses in rheumatoid arthritis [16], and microvascular thrombosis [17]. Furthermore, GLA improves cardiac function, decreases infarcted myocardium, and activates pro-survival signals in mice subjected to a myocardial ischemia-reperfusion injury [17]. In addition, GLA protects human coronary artery smooth muscle cells from H_2_O_2_-induced oxidative stress and inflammation [18]. ROS can upset the balance of the oxidant/antioxidant system by affecting the regulation of enzymatic oxidation, protein oxidation, and lipid peroxidation activities, and ROS can cause tissue damage [19]. In the present study, we found that GLA suppressed ROS production and activated antioxidant enzymes such as SOD. Additionally, GLA inhibited cell death by H/R exposure, as shown by a decrease in PI-positive dead cells. Our results suggested that GLA alleviates H/R-induced oxidative damage in HK-2 cells.

There are various complex negative autoregulatory factors that provide effective control of excessive oxidative stress and inflammation to avoid pathological consequences [20,21]. HO-1 is one of the most critical mechanisms of cell protection that are activated during cellular stress [22]. HO-1 has beneficial effects against oxidative injury, regulation of apoptosis, modulation of inflammation as well as contribution to angiogenesis [23]. In our results, *HO-1* mRNA expression was significantly and markedly elevated by pre-treatment of GLA under H/R-exposure. Nrf2 activates antioxidant response elements (ARE) and increases transcription of Nrf2-regulated genes such as HO-1 or glutathione S-transferase (GST). Our results confirmed that GLA significantly increased the mRNA expression of Nrf2 under H/R-exposure (GLA treatment under H/R vs. H/R). Nrf2/HO-1 system is involvement in development, oxidative stress response, and diseases [23]. Our results also showed that GLA significantly increased the mRNA expression of *AKT* under H/R exposure (GLA treatment under H/R vs. H/R). These findings suggested that GLA activates Akt/Nrf2/HO-1 signaling pathway, leading to inhibit oxidative injury in HK-2 cells.

## 4. Materials and Methods

### 4.1. Chemicals and Cell Culture

GLA, 99.38% purity, was purchased from MedChemExpress USA (Monmouth Jct, NJ, USA) (Batch No. 79AS No. 79498-31-0). The human kidney cell line (HK-2) was obtained from the American Type Culture Collection (Manassas, VA, USA) and maintained in DMEM/F12 medium (Life Technologies, Carlsbad, CA, USA) supplemented with 10% fetal bovine serum (FBS) and 1% penicillin/streptomycin. Cells were incubated at 37 °C in a humidified atmosphere containing 5% CO_2_.

### 4.2. Establishment of H/R In Vitro Model

To stimulate renal I/R injury in vitro, HK-2 cells were cultured in a hypoxic environment with 1% oxygen (O_2_), 94% nitrogen (N_2_), and 5% CO_2_ in modular gas chambers for 24 h, followed by reoxygenation for 2 h in 21% O_2_, 5% CO_2_, and 74% N_2_ incubator at 37 °C. Cells were pretreated with or without GLA for 2 h before H/R exposure.

### 4.3. Cell Cytotoxicity Assay

The cellular injury was assessed by the release of lactic dehydrogenase (LDH) using a commercial kit (TaKaRa, Shiga, Japan) according to the manufacturer’s directions. After incubation with a series concentration of GLA (0, 5, 10, 20, and 40 μM) for 24 h, the culture supernatants were collected.

### 4.4. Cell Viability Assay

Cell viability was evaluated using the cell counting kit-8 (CCK-8) solution assay (Dojindo, Kumamoto, Japan). Briefly, HK-2 cells were seeded at a density of 1 × 10^4^ cells per well in 96-well plates. After various treatments, 10 μL of CCK-8 solution was added to the cells and incubated for 2 h following the manufacturer’s specifications. The cell viability was assessed by measuring the optical density at 450 nm using a microplate reader.

### 4.5. ELISA Assay

After cell culture supernatants were collected for the determination of SOD, cells were lysed in lysis buffer (ATTO Corporation, Tokyo, Japan) for 10 min at 4 °C, and then the cell lysates were collected for the determination of SOD with relevant detection kits (Young Frontier Co., Ltd., Seoul, Korea) per the manufacturer’s instructions.

### 4.6. Quantitative Real-Time PCR

Total RNA was extracted from each cell culture using an RNeasy Mini Kit (QIAGEN, Valencia, CA, USA) and was reverse transcribed into cDNA with a PrimeScript RT Reagent Kit (TaKaRa). The primers adopted for gene expression analysis were the following: AKT1, Hs00178289_m1; *HMOX1*, Hs01110250_m1; *NFE2L2*, Hs00975961_g1; and *GAPDH*, Hs99999905_m1 as housekeeping gene. Analysis was performed using an Applied Biosystems StepOnePlus Real-Time PCR System (Life Technologies).

### 4.7. Dead Cell Assay

After incubation with or without Cd in 24-well plates for 24 h, cells were stained with 8.6 μM propidium iodide (PI; BD Japan, Tokyo, Japan) and 10 μg/mL Hoechst 33342 (Dojindo). Nuclei were visualized using a CKX53 microscope (Olympus, Tokyo, Japan). Dead and viable cells were identified by Hoechst 33342 (blue), whereas dead cells were identified by PI (red).

### 4.8. Statistical Analysis

All values are expressed as means ± SE from three independent experiments. Statistical analysis was performed using a Kruskal–Wallis test with a Dunn’s post hoc comparison test. A *p*-value less than 0.05 was considered significant. All statistical analyses were conducted using GraphPad PRISM, version 4 (GraphPad Software, San Diego, CA, USA).

## 5. Conclusions

We have demonstrated for the first time that GLA, as a novel drug, inhibits oxidative injury in renal proximal tubular cell-line HK-2 cells. Importantly, GLA has almost no cytotoxicity to normal human renal epithelial cells. The above findings provide useful mechanistic information for the treatment of kidney injury, although there is limited information on renal proximal tubular cells and more research is needed using animal experiments.

## Data Availability

Not applicable.

## Abbreviations

GLA	glaucocalyxin A
H/R	hypoxia/reoxygenation
AKI	acute kidney injury
ROS	reactive oxygen species
SOD	superoxide dismutase
LDH	lactate dehydrogenase
MTT	3-(4,5-Dimethylthiazol-2-yl)-2,5-diphenyltetrazolium bromide
HO-1	heme oxygenase-1
Nrf2	nuclear factor erythroid 2-related factor 2
Akt	protein kinase B
